# The Effectiveness of NIV and CPAP Training on the Job in COVID-19 Acute Care Wards: A Nurses’ Self-Assessment of Skills

**DOI:** 10.3390/nursrep13010002

**Published:** 2022-12-27

**Authors:** Stefano Bambi, Eustachio Parente, Yari Bardacci, Samuele Baldassini Rodriguez, Carolina Forciniti, Lorenzo Ballerini, Christian Caruso, Khadija El Aoufy, Marta Poggianti, Antonio Bonacaro, Roberto Rona, Laura Rasero, Alberto Lucchini

**Affiliations:** 1 Department of Health Sciences, University of Florence, 50134 Florence, Italy; 2 Neuroscience—Neurosurgery, Meyer Children’s Hospital, 50139 Florence, Italy; 3 Emergency and Trauma Intensive Care Unit, Careggi University Hospital, 50134 Florence, Italy; 4 Medical and Surgical Intensive Care Unit, Careggi University Hospital, 50134 Florence, Italy; 5 Emergency Department, Careggi University Hospital, 50134 Florence, Italy; 6 Emergency Medical System—AUSL Toscana Centro, 50122 Florence, Italy; 7 Department of Experimental and Clinical Medicine, University of Florence, 50121 Florence, Italy; 8 Hospital Healthcare Management, Careggi University Hospital, 50134 Florence, Italy; 9 School of Health and Sports Sciences, University of Suffolk, Ipswich IP4 1QJ, UK; 10 General Intensive Care Unit, San Gerardo Hospital—ASST Monza, Milano Bicocca University, 20900 Monza, Italy

**Keywords:** CPAP, noninvasive ventilation, education, COVID-19, general ward, nurses

## Abstract

Background: Noninvasive ventilation (NIV) in COVID-19 patients outside of intensive care unit (ICU) settings was a feasible support during the pandemic outbreak. The aim of this study was to assess the effectiveness of an “on the job” NIV training program provided to 66 nurses working in 3 COVID-19 wards in an Italian university hospital. Methods: A quasi-experimental longitudinal before–after study was designed. The NIV Team education program, provided by expert ICU nurses, included: 3 h sessions of training on the job during work-shifts about the management of helmet-continuous positive airway pressure (CPAP) Venturi systems, and NIV with oronasal and full-face masks. An eleven-item “brief skills self-report tool” was administered before and after the program to explore the perception of NIV education program attendees about their level of skills. Results: In total, 59 nurses responded to the questionnaire. There was an improvement in the skill levels of the management of Helmet-CPAP (median before training 2, inter-quartile range (IQR) 0–6; median after training 8, IQR 3–9; *p* < 0.0001), and mask-NIV (median before training 2, IQR 0–6; median after training 8, IQR 3–9; *p* < 0.0001). Conclusions: Training on the job performed by expert ICU nurses can be a valuable and fast means to implement new Helmet-CPAP and mask-NIV skills outside of ICUs.

## 1. Introduction

In the last 15–20 years, NIV support for acute patients has been “exported” outside the intensive care unit (ICU) “walls”, across high dependency units (HDUs) into general wards and finally outside the pre-hospital emergency settings [1,2]. The application of NIV on ARF patients in general medical wards is associated to high success rates, up to 80.9% [3].

During the COVID-19 pandemic BiPAP and CPAP supports have been largely employed to prevent intubation [4], reducing mortality rates, and decreasing the admission rates in ICU [5,6]. A recent systematic review showed that delivering NIV support in COVID-19 patients outside intensive care settings was a feasible strategy during the high demand of ventilatory support provided in pandemic outbreak [7]. The rate of NIV failure was 26% (CI 95%: 21–30%) [7]. The use of NIV or CPAP supports in COVID-19 general ward patients not appropriate for admission to intensive care unit was retrospectively associated to a survival rate of 50% [8]. Behind these data, there were some challenging issues that nurses had to deal with. On one hand there was the transformation of medical general wards in acute COVID-19 wards, by the introduction of new ventilators and CPAP systems. On the other hand, these “new” wards were filled with many newly hired nurses without skills to manage NIV technologies and the patients–interfaces–NIV system interactions [9,10,11]. In fact, the application of NIV requires special skills and competencies related to set-up and management of ventilators and high-flow devices, achieving adequate levels of patients’ compliance to the ventilation, complication’s prevention (such as discomfort, pain and interface-related pressure injuries), and optimal troubleshooting about patient–ventilator interaction. Moreover, nurses should be aware of the physical and communication needs of patients during NIV support, and especially the potential condition of anxiety, loss of control feeling and panic that the diverse typologies of interfaces could generate [12,13].

Twenty-four-seven availability of healthcare personnel with adequate skills to manage NIV patient is the clinical key to reach positive outcomes in general wards [14]. Scientific literature frequently showed the lack of doctors’ and nurses’ knowledge and competence about NIV [15] is the reason behind its scarce application in clinical settings [16,17,18]. Data from Brazil reported that only 30% of nurses have a know-how to initiate NIV support [19] and that 77% of medical personnel did not know the NIV initiation criteria [20]. The 88% of the surveyed personnel declared not to know how to set ventilator’s alarms and identify patient–ventilator dissynchronies [20]. Furthermore, a European survey published in 2014 showed that in 41% of enrolled wards there were less than five nurses with adequate skills for NIV application in acute respiratory failure patients [21]. Nurses seem to have low levels of knowledge and confidence in NIV support [19]. Few nurses know how to choose the right size of NIV facial masks (35%), to identify patient–ventilator dissynchronies, and to change the ventilator’s setting parameters (38%) [22]. A Delphi study published in 2012 identified nine core objectives for NIV education programs focusing on modes of ventilation, interfaces, indications and contraindications, evidence for application in various clinical conditions, set-up and initiation of NIV, troubleshooting, monitoring, failure and success indicators, and complications [23]. An adequate NIV educational program should be based on the educational needs assessment, the availability of updated guidelines, clinical procedures, and equipment. The NIV educational program should also be flexible and adaptable to the different needs of multidisciplinary team members and their individual experience levels [24]. NIV requires an education and training time directly proportional to the complex technologies used for delivering [14]. In fact, NIV failure rates are closely related to the levels of training and experience of the healthcare personnel (e.g., the application of an adequate size interface and an appropriate management can reduce air-leaks and risk of unsuccessful outcomes) [25].

Theoretical knowledge is crucial for education for every kind of issue. However, NIV education always requires a clinical practice integration to be really effective. Simulation is a promising educational and training method to acquire adequate competencies for managing NIV patients. Some studies showed that low fi manikin or off-screen feedback sessions can be helpful to reduce the time needed for becoming confident with NIV and patients’ management [26,27]. Currently, NIV education performed through high-fidelity simulation is still not largely widespread [28]. An optimal NIV educational program should be composed of theoretical lectures for a third, learner–teacher interaction for a fifth, and the remaining half of time spent for training on the job. Beyond the basis of NIV management, the educational program should focus on critical issues such as nasogastric tube insertion indication, active humidification settings, intra-hospital transport, the use of high-flow oxygen therapy during the breaks from NIV treatment cycles, and the management of patients “NIV-dependent”, which are patients with a very low respiratory reserve, that need to be maintained for long period under NIV support. As a general principle, one useful experience during the training course, is to don a helmet or a mask and experience NIV support and feel all the sensations and discomfort suffered by their future patients [29].

Beyond the experiences of educational programs reported by some authors and the experts’ recommendations, evidence about the effectiveness of different methods in teaching the management of patients undergoing NIV is still lacking [29]. Moreover, no data about teaching and learning NIV skills in general wards during COVID-19 pandemics were still published in scientific literature.

Based on the above-mentioned issues, this study aimed to demonstrate the effectiveness of an “on the job” NIV training program provided to nurses working in COVID-19 medical wards during the second wave of the COVID-19 pandemic in an Italian university hospital.

## 2. Materials and Methods

### 2.1. Design

A quasi-experimental longitudinal single cohort before and after study was designed.

### 2.2. Sample and Setting

The study involved all the 66 nurses working in three acute medical wards of Careggi University Hospital converted to COVID-19 general wards at the beginning of the second wave of pandemic in October 2020.

The teams of these wards (anonymously called “1”, “2”, “3”) were numerically enhanced with nurses coming from other hospital clinical settings temporarily in stand-by, and many newly hired nurses to consent adequate times of break during the work-shifts with personal protective equipment (PPE). Moreover, these 3 wards were equipped with new ventilators and systems to deliver NIV and CPAP to COVID-19 patients. Therefore, a large educational need emerged by nurses, to manage patients undergoing to these respiratory supports.

### 2.3. Procedure

The director of healthcare professions department of Careggi university hospital formed a group of four intensive care unit nurses with high skills in noninvasive ventilation and CPAP. This group, called “NIV Team”, had the task to plan an on-the-job training program on NIV and CPAP for nurses.

The educational program was based on the hospital official procedure for noninvasive ventilation for patients in general ward, which was specifically updated for the safety issues related to the caring of COVID-19 patients.

The NIV Team education program included: 3 h sessions of training on the job during morning and afternoon work-shifts; the production of simple charts and videos containing the setting-up of the different Helmet-CPAP Venturi systems; and the breathing circuit of NIV performed with oronasal and full-face mask on various mechanical ventilators. Furthermore, a reserved email address was provided to collect and answer the requests of advice by nurses and other healthcare professionals in need.

Lastly, two brief checklists for NIV and CPAP management were drafted and diffused among all the nurses involved in the training program and the physicians working in the three wards.

The NIV Team training on the job program (composed of 2 training sessions) was provided between November 2020 and January 2021, during the morning and afternoon shifts. The aim of NIV Team was to guarantee the presence of at least one expert nurse to perform training-on-the-job session, until every nurse working in the ward had attended the 2 training sessions, giving immediate practical feedback to the trainer about the level of learning obtained.

The educational and training contents of the program were: “refreshing about the use of oxygen therapy, especially through high flow nasal cannula”; “principles of CPAP and NIV supports”; “set-up of Helmet-CPAP through Venturi systems and patients’ management”; “set-up of NIV (Spontaneous-Time Mode; Pressure Support Ventilation) with oronasal and full-face masks, and patients’ management”.

The training was fitted on the educational needs of every single nurse at the bedside following these steps: (1) basic principles of oxygen-therapy and NIV were refreshed and explained; (2) demonstration of NIV and CPAP systems were performed; (3) training of the attendee was carried on until a positive feedback about the acquired skill was obtained; (4) adequate time for answers and questions was provided; (5) training on NIV and CPAP system troubleshooting was performed at the bedside.

Many models of intentional leak NIV ventilators and ICU ventilators were employed in the COVID-19 general wards, increasing the need for focused training sessions as well as for medical personnel. However, the consultations offered to physicians were performed occasionally and were not included in a structured educational program.

### 2.4. Data Collection and Instrument

An essential “brief skills self-report tool” was designed by the members of NIV Team to explore the perception of NIV education program attendees about their level of skills on the set-up and management of patient undergoing to Helmet-CPAP and NIV. The tool was composed of 11 items investigating the rate of some “core” learned interventions, and 2 items requiring the self-perception of the NIV and CPAP overall management skills levels, before and after the education program. The Items included in the before and after “brief skills self-report tool” with their abbreviations are listed below:

- Maintenance frequency of patients with CPAP-helmet or NIV in Fowler position (Frequency Fowler position).
- Check frequency of the Helmet-CPAP system and/or NIV, and surveillance of patients during the treatment (NIV patients check frequency).
- Frequency of respiratory rate measurement in patients with COVID-19 (RR assessment frequency).
- Frequency of application of 24 h expiring HEPA filters on the exit port of the CPAP-Helmet (HEPA 24-h application frequency).
- Check frequency of pulse-oximetry during helmet CPAP removal pauses or NIV mask breaks (Check SpO_2_ frequency).
- Frequency of setting up of the Helmet-CPAP to the patients by 2 healthcare professionals (Frequency of helmet set-up by 2 nurses).
- Frequency of assessment of the Helmet-CPAP system effective performance by appreciating with gloved hands the presence of continuous gas flow leaving the PEEP valve during inspiration and exhalation (CPAP system working check frequency).
- Frequency of use of the Helmet armpits outside the arms of the patient, placing weights (sandbags) to maintain the system in place and limit air leaks [10] (Helmets armpits outside frequency).
- Frequency of use the “off” function instead of “standby” to pause the NIV mask session delivered with a single tube intentional leaks NIV ventilator (Off ventilator frequency).
- Frequency of use of traditional oxygen therapy systems (reservoir masks, standard masks, Venturi masks, nasal cannula) during breaks from Helmet-CPAP and/or NIV mask (O_2_ therapy for NIV breaks frequency).
- Frequency of autonomously setting up of a single tube intentional leaks NIV ventilator (Autonomous set-up of NIV circuit frequency).
- Self-perception of skills levels in patient management with Helmet-CPAP (Helmet-CPAP skill levels).
- Self-perception of skills levels in patient management with NIV mask through single tube intentional leaks NIV ventilator (NIV skills levels).

The choice to ask the “frequency” of practicing the learned core intervention was made with the aim of obtaining an indicator of skills acquired as “less subjective” as possible. All the items were evaluated by an eleven-point numerical scale (from 0—“never” or “absent”, to 10—“always” or “total”).

The brief skills report tool showed high internal consistency (Cronbach Alpha values: 0.977 for all the tool items; 0.930 for the before training tool items; 0.982 for the after-training tool items).

Demographical data were also collected, such as age, gender, ward, total length of service as nurse, current ward length of service, and previously NIV courses attendance.

The before and after “brief skills self-report tool” was transferred on a Google Form sheet and administered to the attendants after a week after the end of the education program. Data were collected on an xls sheet and were stored in personal computer accessible only through a password known by the researchers.

### 2.5. Statistical Analysis

The collected data underwent a preprocessing (recoding) phase, and after the assessment of the not-normality of distribution through the Shapiro–Wilk test, a descriptive analysis was performed using median and quartile values. Inferential and explorative statistics were performed by non-parametrical statistics using the Wilcoxon Signed Ranks Test for paired groups to highlight the differences between the perception of skills levels before and after the training on the job, while the analysis of the skills levels inter-groups was performed through the Kruskal–Wallis Test. Categorical and binomial data were explored using Chi Square test. IBM SPSS 22 and GraphPad Prism 5 were used for statistical analysis.

### 2.6. Ethical Issues

The electronic form containing the NIV “brief skills self-report tool” was administered maintaining the anonymity, via the informal WhatsApp chat of the three medical wards involved by the NIV Team training program. The questionnaire did not contain any item requiring data that could identify either the single respondents or the ward of affiliation. Moreover, the data set was stored and protected following the local institutional procedures, and the data analysis was performed in an aggregate way according to the national privacy regulation.

The study was conducted as a part of the outcome evaluation of the training on the job program, after having obtained the consent of the healthcare professions direction office of the hospital.

According to local ethical committee (EC) guidelines, the administration of questionnaires to healthcare workers did not require EC formal approval.

## 3. Results

### 3.1. Characteristics of Participants

In total, 59 of the 66 nurses that attended the NIV education program (89.4%) responded to the “brief skills self-report tool” (ward 1: N.16, 27.1%; ward 2: N.23, 39%; ward 3: 20, 33.9%); 48 (81.4%) were female. The median age of respondents was 41 years (IQR 34.5–49; range 22–52). The median total length of service as staff nurse and length of service in the ward of current assignation were 15 years (IQR 10–23; range 2–38) and 6 years (IQR 4–16.5; range 1–20), respectively. None of the respondents had any post-graduate critical care or emergency courses certifications.

The respondents’ characteristics, distributed by the three wards of assignation, are shown in Table 1. Although ward 3 accounted for a median age and length of service slightly lower than wards 1 and 2, the differences were not statically significant.

### 3.2. Results of the “Brief Skills Self-Report Tool”

**Figure 1:**
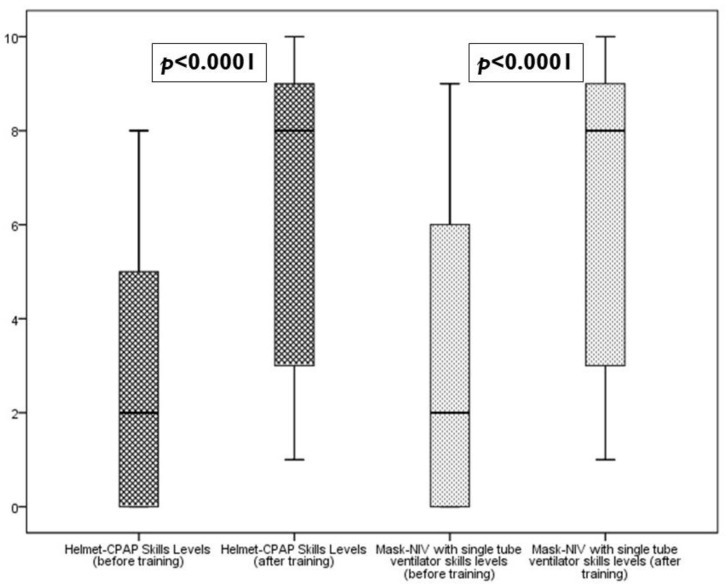
Comparison of Helmet-CPAP and NIV skill levels before and after the training (total sample—59 nurses).

After the training on the job, the totality of nurses who filled the questionnaire showed a statistically significant increase in their perception about skill level in management patients undergoing Helmet-CPAP (median before training 2, IQR 0–6, range 0–9; versus median after training 8, IQR 3–9, range 1–10; Wilcoxon Signed Ranks Test *p* < 0.0001) and mask-NIV with single tube ventilator (median before training 2, IQR 0–6, range 0–9; versus median after training 8, IQR 3–9, range 1–10; Wilcoxon Signed Ranks Test *p* < 0.0001) (Figure 1).

**Figure 2:**
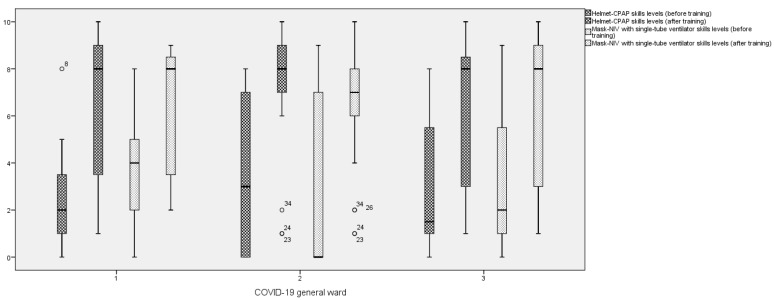
Comparison of Helmet-CPAP and NIV skill levels before and after the training by wards 1, 2, and 3.

The results of the items related to the frequency of practicing the CPAP and NIV skills before and after the education program in the total sample of nurses are shown in Table 2. The largest differences in the median values before and after the training were recorded in the items related to: (1) the application of HEPA filters at the expiratory port of the CPAP-Helmet, (2) the control of the CPAP systems effective performance, (3) the use of sandbag weights to maintain the helmet armpits outside the arms of the patient, the set-up of a NIV ventilator breathing circuit, and (4) the use of the ventilators’ off function instead of the stand-by to avoid aerosolization exposure during the removal of patients’ mask for NIV-breaks. There were no statistically significant differences among the median values of the tool after the training by the three COVID-19 wards, indicating that the aims of the educational programs were homogeneously reached (Table 3). All the COVID-19 wards recorded large differences between the skills levels perceptions of nurses related to CPAP and NIV management before and after the training (Figure 2).

Concerning risk control, periodical contacts and visits by the NIV Team members were performed across the three wards, and up until now, no adverse events related to the introduction of CPAP and NIV technologies were detected after the education program.

## 4. Discussion

According to a recent expert consensus, NIV and CPAP should be considered in the support of COVID-19 patients with hypoxemic and hypercapnic respiratory failure showing an increased work of breathing [30]. The benefit of NIV was largely shown in a recent review reporting that in 5120 COVID-19 patients with noninvasive respiratory support (High Flow Nasal Cannula, CPAP, or NIV), the intubation rate was 37% (1880) and survival was 78% (4669) [31].

In a survey among respiratory therapists in Saudi Arabia, the lack of training was one of the main barriers (43%) to ventilatory support management of COVID-19 patients, behind staff shortage, PPE shortage and high workload [32]. As a recent consensus paper stated, healthcare professionals with high expertise in management NIV equipment and patient–ventilator interaction are a key factor to achieve patients’ positive outcomes [33] especially in long periods of NIV support application [34]. However, evidence on the best modalities to accomplish educational programs is still lacking [33]. The standards for education in NIV published in 2019 recommended the use of “simulation-based teaching” in NIV, and the need to provide “repetitive sessions depending on the degree of expertise” of the trainees [33]. The training program reported in this paper has moved one step ahead of these recommendations, because the training was provided directly in the clinical setting of the trainees and the duration of the sessions were variable on the single learned feedback.

Authors state that a reasonable NIV educational program time should be made of lectures for 30%, question time for 20%, and training for 50% [33]. The present paper reported on an experience during an emergency, with the aim of providing, in the fastest way possible, a core set of skills and competencies to be immediately applied at patients’ bedsides. Therefore, about 80% of the time was spent on the hands-on training, while an essential theoretical refreshing was being provided. In fact, all the education sessions were performed inside the COVID-19 wards, and the speaking for long time could be exhausting for the trainers, due to the presence of personal protective equipment (PPE).

Ramirez et al. (2020) reported that the 58% of patients with CPAP support in COVID-19 internal medicine wards underwent at least a session of prone positioning [35]. The NIV Team training on the job program did not include any specific issue related to the nursing during NIV or CPAP patients undergoing prone position, even if the contents of this educational program covered all the aspects related to the monitoring and management of patients’ interactions with the noninvasive respiratory supports, fitting also prone-positioned patients.

Recent research performed on CPAP support applied in COVID-19 wards found high discontinuation rates of CPAP among patients, probably due to an increasing burden of treatment that deserves to be investigated [36]. This potential element should also be taken into account in designing educational programs for nurses because the endurance of patients undergoing to CPAP and NIV support is one of the keys for the treatment success.

Robinson et al. (2021) reported the implementation of CPAP delivered using domiciliary ventilators in an Infectious Disease unit in a UK NHS hospital during the first wave of the COVID-19 pandemic [37]. The ward staff were adequately trained for the use of CPAP home ventilators and supported by the local critical care team. The authors reported a median CPAP length of treatment of 4 days (range 2–5), and 65% of patients (17/26) that avoided the endotracheal intubation were treated [37]. Even if the experience of NIV Team training on the job program lacked in the measures of similar hard outcomes, the reported results show that the aim of education was fully reached because all of the expected behaviors were improved.

### Limitations

This study was affected by some limitations due to the “hard time” when it was conducted. The COVID-19 pandemic “healthcare emergency” required a rapid and effective response to new needs coming from the lack of skills and knowledge about new technologies introduced in “usual” low-complexity care settings. Therefore, the choice of the study design was inevitably a quasi-experimental research, with a higher exposure to unobservable sources of bias and confounding (e.g., self-selection bias, differential motivation, previous work experience in critical care settings) than an experimental design, thus limiting the evidence emerging from this study

The evaluation of acquired skills of the nurses attending to the educational program was performed through a self-report tool. Moreover, this tool, even if very simple, did not undergo any validity tests. Finally, we cannot exclude a recall bias (especially for the pre-training item responses) due to the fact that the administration of the tool occurred only after the educational program was termed. Moreover, we could not prevent the possibility of some contaminations among the samples during the filling of the questionnaires; however, this potential bias could have occurred only inside the single wards, not between a ward and another. Thus, the lack of adoption of a validated tool for competency assessment remains probably the main limitation of this study.

The only other mean of skills assessment used by NIV Team was the feedback provided by the trainees during the educational sessions.

No other objective outcomes of this education program could be collected and measured due to the wards’ difficulties

## 5. Conclusions

Educational programs based on training on the job performed by expert professionals can be a valuable and fast means of implementing or updating new Helmet-CPAP and mask-NIV patients’ management skills among nurses outside of intensive care settings.

Objective quality indicators as an observational performance checklist or ex post exams to evaluate the maintaining of acquired skills should be implemented.

Since the reconversions of some wards to no-COVID-19 wards, the maintenance of CPAP and NIV competencies and skills should be conducted through refreshing sessions in a high-fidelity-simulation environment and evaluated by study designs with robust internal validity.

## Figures and Tables

**Table 1:** Demographical characteristic of the respondents by the ward of current assignation.

Variable	Ward 1	Ward 2	Ward 3	*p*
Gender Female—*n*. (%)	14 (87.5)	20 (87)	14 (70)	0.276 ^a^
Age—Median (IQR; range)	45 (38.5–49.75; 26–52)	42 (33–50.25; 24–51)	36 (29–48; 22–50)	0.113 ^b^
Total length of service as staff nurse—Median (IQR; range)	19.5 (10–21.25; 6–28)	16.5 (9.5–26.5; 2–30)	13 (10–22; 4–38)	0.819 ^b^
Length of service in the ward of current assignation—Median (IQR; range)	9 (4.5–17.25; 2–19)	12 (4.5–20; 1–20)	6 (1.5–8; 1–20)	0.137 ^b^
NIV/CPAP education courses in the last 5 years	7 (41.2)	11 (45.8)	15 (60)	0.428 ^a^

Legend: ^a^—Chi square test; ^b^—Kruskall–Wallis test.

**Table 2:** Frequency of practicing the skills before and after the training on the job in the total sample (59 nurses).

Item (Abbreviated)	Before Training Median (IQR; Range)	After Training Median (IQR; Range)	Wilcoxon SIGNED Rank Test *p*
Frequency Fowler position	6 (2–9; 0–10)	9 (3–10; 2–10)	<0.0001
NIV patients check frequency	5 (2–8; 0–10)	9 (3–10; 2–10)	<0.0001
RR assessment frequency	5 (2–7; 0–10)	7 (3–10; 0–10)	<0.0001
HEPA 24-h application frequency	3 (0–7; 0–10)	9 (3–10; 1–10)	<0.0001
Check SpO_2_ frequency	5 (3–9; 0–10)	9 (3–10; 2–10)	<0.0001
Frequency of helmet set-up by 2 nurses	5 (2–8; 0–10)	8 (3–10; 1–10)	<0.0001
CPAP system working check frequency	1 (0–3.25; 0–10)	7 (3–10; 0–10)	<0.0001
Helmets armpits outside frequency	0 (0–0; 0–6)	7 (2–9; 0–10)	<0.0001
Off ventilator frequency	2 (0–6; 0–10)	8 (3–10; 0–10)	<0.0001
O_2_ therapy for NIV breaks frequency	8.5 (3–10; 0–10)	9 (3–10; 2–10)	<0.0001
Autonomous set-up of NIV circuit frequency	2 (0–6.25; 0–10)	6 (2.5–9; 0–10)	<0.0001

**Table 3:** Frequency of practicing the acquired skills after the training on the job by wards 1, 2, and 3 (59 nurses).

Item (Abbreviated)	Ward 1 Median (IQR; Range)	Ward 2 Median (IQR; Range)	Ward 3 Median (IQR; Range)	Kruskall Wallis Test *p*
Frequency Fowler position	9 (3.5–10; 2–10)	9 (8–10; 2–10)	7.5 (2.25–10; 2–10)	0.270
NIV patients check frequency	8.5 (3.5–10; 2–10)	9 (8–10; 2–10)	7.5 (3–10; 2–10)	0.509
RR assessment frequency	6.5 (3–9.75; 1–10)	8 (3–10; 0–10)	8 (2.25–10; 2–10)	0.970
HEPA 24-h application frequency	9 (3.75–10; 2–10)	9 (8–10; 2–10)	8.5 (3–10; 1–10)	0.476
Check SpO_2_ frequency	8 (3.5–9.75; 2–10)	9.5 (7.25–10; 2–10)	8 (3–10; 2–10)	0.414
Frequency of helmet set-up by 2 nurses	8.5 (3.75–10; 2–10)	8 (6–9; 2–10)	7.5 (3–10; 1–10)	0.641
CPAP system working check frequency	8 (3.25–9.75; 1–10)	7 (2–10; 0–10)	7 (3–10; 2–10)	0.755
Helmets armpits outside frequency	7.5 (4–8.75; 2–10)	7 (2–9; 0–10)	7 (2–10; 0–10)	0.764
Off ventilator frequency	9 (3–10; 1–10)	8 (2–10; 0–10)	8 (3–10; 2–10)	0.674
O_2_ therapy for NIV breaks frequency	9 (4–10; 2–10)	9 (8–10; 2–10)	7 (3–10; 2–10)	0.562
Autonomous set-up of NIV circuit frequency	7.5 (3–9.25; 2–10)	6 (1–8; 0–10)	3 (2.25–9; 0–10)	0.492
Helmet-CPAP skill levels	8 (3.25–9; 1–10)	8 (7–9; 1–10)	8 (3–8.75; 1–10)	0.466
NIV skills levels	8 (3–9; 2–9)	7 (5.5–8.25; 1–10)	8 (3–9; 1–10)	0.977

## Data Availability

Data of this study are available on request to the corresponding author.
